# Non-Response of Epstein–Barr Virus-Associated Breast Cancer after Primary Chemotherapy: Report of Two Cases

**DOI:** 10.3390/pathogens12121387

**Published:** 2023-11-24

**Authors:** Ewgenija Gutjahr, Carlo Fremd, Johanna Arnscheidt, Roland Penzel, Jürgen Wacker, Peter Sinn

**Affiliations:** 1 Department of General Pathology, University Hospital, 69121 Heidelberg, Germany; 2 Department of Medical Oncology, National Center for Tumor Diseases, University Hospital and German Cancer Research Center (DKFZ), 69121 Heidelberg, Germany; 3 Department of Obstetrics and Gynecology, Fuerst-Stirum-Hospital, 76646 Bruchsal, Germany

**Keywords:** breast cancer, EBV, therapy resistance

## Abstract

Based on epidemiological evidence and molecular findings, a possible association of Epstein–Barr virus (EBV) with the carcinogenesis of breast cancer has been described. However, the frequency of EBV in breast cancer and the role of EBV regarding tumor progression or therapeutic results is largely unexplored. Here, we report on two cases of advanced, lymph node-positive invasive breast cancer of no special type (NST), histologically showing no clinical or histological evidence of tumor regression as an equivalent of a lack of response to primary systemic therapy. Both tumors were considered to be EBV-associated due to their positivity in EBV-encoded RNA (EBER) in situ hybridization (ISH) and their immunoreactivity against EBV Epstein–Barr nuclear antigen 1 (EBNA1). We hypothesize that the unusual non-response to chemotherapy in these cases of breast cancer classified as triple-negative and HER2-positive may be linked to the EBV co-infection of tumor cells. Therefore, EBV tumor testing should be considered in patients with breast cancer presenting with resistance to chemotherapy. This hypothesis may provide a new aspect in the context of EBV-associated mechanisms of tumor progression.

## 1. Introduction

The fact that viruses can contribute to and accelerate the multistep oncogenesis of several tumor entities is broadly accepted today. Among carcinogenic agents, Epstein–Barr virus (EBV) is regarded by the International Agency for Research on Cancer (IARC) as one of the most important viral agents causing malignancy in humans [1]. Annually, it is estimated to be associated with some 120.000 cases of tumor diseases worldwide [2]. Based on the epidemiological evidence and EBV detection, EBV infection is linked to the carcinogenesis of several types of malignancies, namely nasopharyngeal and gastric cancer as well as lymphoma [3,4]. Additionally, cases of leiomyosarcoma have been reported in the immunocompromised host [5,6], and the role of EBV has been discussed in oral squamous carcinoma [7].

In breast cancer (BC), the evidence regarding EBV-associated tumorigenesis is controversial. The association of BC with EBV was reported almost 30 years ago [8]. In this landmark paper, the authors analyzed breast cancer DNA in parallel with blood samples obtained from the same patients and detected the EBV genome in some 20% of the cases of breast tumors. Further, they speculated that EBV may account for the “diversity and the sometimes unexpected behavior” of breast cancer. Since then, the EBV genome has repeatedly been detected in breast cancer by means of PCR and in situ hybridization in various countries [9,10,11,12,13,14,15,16,17,18,19,20,21,22,23,24,25,26,27,28,29,30,31]. In some studies, EBV-positive lymphocytes that may occur in tumor stroma were not clearly excluded [32,33,34]. No association between the EBV genome and breast cancer tumor cells could be identified in the two case series, and in these studies, the causative role of EBV was disputed [35,36]. In a recent meta-analysis, the odds ratio of EBV-positive breast cancer vs. benign breast controls was calculated as 4.75 [37]. In another meta-analysis, based on 24 case-control studies, the prevalence of EBV was 731 (30.4%) in 2402 breast cancers, as compared to 52 (7.5%) in 1044 normal and benign breast tissue controls [38]. Taken together, consistent data point to a higher prevalence of EBV infection in breast cancer compared to controls.

The evidence regarding the role of EBV in breast cancer has been reviewed In three articles [39,40,41,42]. Farahmand et al. concluded that because of the high seroprevalence of EBV in the normal human population (85.3% in the UK [43], 66.5% in the US [44]), it may be difficult to assess any causal link between the presence of anti-EBV antibodies and breast cancer risk, but noted that the published data were supporting the hypothesis that EBV-infection is a risk factor for breast cancer [40]. Similarly, Shechter et al. classified the available evidence as “not definitive, strong evidence”, mainly because of the higher prevalence of EBV positivity in breast tumor tissue compared to controls [42]. Lawson pointed out that in contrast to other virus-associated malignancies, the prevalence of breast cancer is not increased in immunocompromised patients [45], and therefore, tumor viruses, including EBV, may not be responsible for the initiation of breast cancer but may play a secondary role in this disease [41].

Although the link between EBV and breast cancer still is not well understood, we present two clinical cases of EBV-associated breast cancer, suggesting that tumor-promoting mechanisms of EBV could be important in the context of non-response to chemotherapy.

## 2. Materials and Methods

### 2.1. Immunohistochemical Staining

Formalin-fixed and paraffin-embedded (FFPE) tumor tissue sections were deparaffinized, pretreated by EDTA unmasking solution (pH = 9 Dako, S2367) for 45 min at 90 °C, followed by hydrogen peroxide blocking for 7 min and NGS blocking for another hour. Sections were incubated with primary antibodies EBNA1 (Millipore MABF-2800-25 UG, rat, overnight at 1:100) and EBV LMP1 (Abcam, ab78113, mouse, 1 h at 1:200), followed by HRP-coupled anti-mouse secondary reagent (Enzo Life Sciences, Farmingdale, NY, USA), DAB substrate as chromogen (Agilent Dako, Santa Clara, CA, USA) and counterstaining. Sections were scanned by Aperio AT2 (Leica Biosystems, Wetzlar, Germany) for histologic evaluation.

### 2.2. EBV-Encoded RNA (EBER) In Situ Hybridization (ISH)

EBER-ISH using ZytoFast® kit (T-1063-40, Zytovision, Bremerhaven, Germany) in combination with the ZytoFast® Digoxigenin-labeled EBV Probe (Ref. T-1114-400, Zytovision) was performed on FFPE tissue sections in accordance with the manufacturer’s instructions to detect the expression of EBER-1 and EBER-2 in tumor tissue. On-slide controls (MB-CC VIR, Zytomed Systems, Berlin, Germany) were used to validate the results of the in situ hybridization.

## 3. Results

### 3.1. Case 1

**Figure 1:**
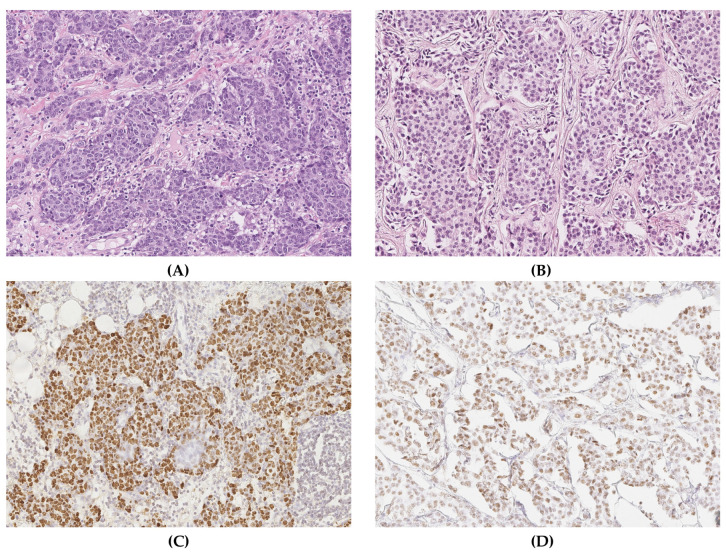
(**A**,**B**) Conventional histopathology (H&E stain) of both cases after chemotherapy and (**C**,**D**: H&E, 20×) the results of EBER-ISH. A solid growth pattern is seen in both tumors, but with high nuclear pleomorphism in case 1 (**A**) and moderate pleomorphism in case 2 (**B**). (**C**,**D**) EBER-ISH reaction shows positive signals in the great majority of tumor cells in both cases.

**Figure 2:**
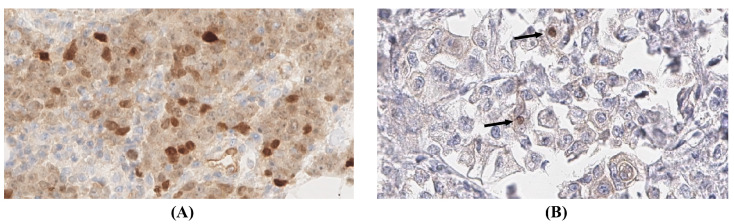
(**A**,**B**) Results of immunohistochemical staining with EBNA1 [30×]. A patchy pattern of nuclear positivity against EBNA1 within tumor cell nuclei is evident in case 1 (**A**), and strongly positive nuclei in fewer tumor cells (→) are seen in case 2 (**B**).

Clinical and treatment data have been summarized in Table 1. On core needle biopsy, the tumor was characterized as triple-negative, highly proliferating (Ki-67 index: 90%), poorly differentiated invasive breast cancer of no special type (NST, G3). After completion of neoadjuvant chemotherapy and mastectomy, no pathologic evidence of tumor regression was evident that could be attributed to the systemic therapy (Figure 1A). Because of the history of EBV-positive nasopharyngeal carcinoma, the tumor was tested for the presence of EBV using chromogenic in situ hybridization (EBER-CISH). Strong nuclear hybridization signals were detected in about 70% of tumor cell nuclei (Figure 1C). On IHC for EBNA1 antigen, a moderate to strong nuclear expression was observed in a heterogenous pattern (Figure 2A,B). Clinically, multiple local and distant lymph node metastases, as well as diffuse bone metastases, were observed four months after the diagnosis of breast cancer, thus attesting to a progressive disease despite the therapy.

### 3.2. Case 2

Clinical and treatment data have been summarized in Table 2. On core needle biopsy, the tumor was characterized as moderately differentiated, invasive breast cancer of no special type (NST, G2; ER+/PR+/HER2+). Following neoadjuvant chemotherapy and breast-conserving therapy, no evidence of tumor regression was evident on the resection specimen (Figure 1B). Because of the lack of histological tumor regression, EBER-ISH was performed on the tumor tissue after chemotherapy, showing convincing positive nuclear hybridization signals (Figure 1D). Also, in this case, an immunohistochemical reaction against the EBNA1 antigen was positive in a similar pattern, as observed in case 1 (Figure 2B).

## 4. Discussion

In the last decades, significant improvements have been made in achieving complete remission (pCR) following preoperative systemic treatment in breast cancer [46,47]. Meanwhile, a pCR rate of roughly 60–80% can be achieved for triple-negative and HER2-positive breast cancer. Thus, cases showing non-response or tumor progression under neoadjuvant chemotherapy (NACT) are rare, with a rate of about 3% in a large case series [48]. Progressive disease (PD) in NACT is characterized by the increase in tumor size or the development of new tumor lesions in the breast, lymph nodes, or distant sites [49]. Known risk factors for tumor progression under NACT are African American ethnic origin as well as clinical and histopathological tumor stage (according to TNM as well as to AJCC classification) [48].

Possible mechanisms of resistance to chemotherapy or targeted therapy leading to tumor progression have been grouped as alterations in the target or in the targeted pathway, activation of alternative pathways, microenvironment-mediated resistance mechanisms, and others, such as metabolic pathways [50,51,52]. They may be facilitated by intra-tumor heterogeneity and clonal diversity, playing an important key role in the evolution of cancer [53,54]. Specifically, in HER2-positive disease, several mechanisms have been associated with resistance to anti-HER2 therapy in vitro and in vivo. These include expression of the truncated HER2 receptor fragment p95 leading to aberrations in HER2 signaling, aberrant downstream signaling caused by activating mutations of phosphatidylinositol 3-kinase (PIK3CA) gene, increased signaling through other HER family members, and prevention of cell cycle arrest [55,56]. Also, mutation of the HER2 gene may lead to resistance, depending on the type of anti-HER2 therapy [57]. In triple-negative breast cancer, acquired resistance to targeted therapy is a frequent phenomenon that is associated with the mechanisms of action of different kinds of targeted therapies in TNBC [58,59] but is also linked to molecular subtypes of TNBC [60]. Hence, the identification of potential mechanisms responsible for primary or acquired resistance to chemotherapy or targeted therapy is important to improve and personalize the therapeutic strategies.

Here, we have presented two cases of triple-negative or HER2-positive breast cancer with a lack of tumor regression or progression under chemotherapy. In both cases, neither a reduction in tumor size nor histopathological evidence of tumor regression was evident, as defined in the literature [61]. The detection of EBV markers by ISH and IHC in the tumor cells may, amongst other mechanisms, imply a role of EBV in the resistance of tumor cells to chemotherapeutic agents. Also, in both cases, no special histologic features were detectable that would have suggested in-breast metastases originating from EBV-related cancer elsewhere. In the first case, a history of EBV-associated nasopharyngeal carcinoma was given that clinically had a full response to neoadjuvant chemotherapy and was not operated upon. This, and the different histological tumor types of histopathology of nasopharyngeal carcinoma, indicates that the breast cancer was, in fact, a secondary EBV-related malignancy in this patient.

Pathogenic pathways of EBV activity in malignancies, as described in malignant lymphoma and in nasopharyngeal carcinoma, include blocking apoptosis and promoting tumor proliferation as key factors in EBV-associated tumorigenesis [62,63,64]. Apoptosis, i.e., programmed cell death, plays a significant role in breast cancer. Aberrations in apoptotic pathways are related to tumorigenesis and tumor progression and growth, as well as regression response to chemotherapy, radiotherapy, and endocrine treatment [65,66,67]. The evidence that EBV can regulate apoptosis is compelling [68]. Several mechanisms have been shown to play a part in this, including blocking PKR phosphorylation [69] and other mechanisms [70,71,72]. BARF1 plays a role by activating BCL-2 [73] and also induces cell cycle activation [74,75]. In addition, it was shown that the expression of two viral homologs of BCL-2 is important for providing anti-apoptotic signals in newly infected B-cells. In this study, EBV-infected cells immediately underwent apoptosis without these BCL-2 homologs, but these proteins were no longer essential once latent infection was established [76]. These mechanisms have already been shown to be relevant for chemotherapy resistance in vitro [77] and may be important in conferring resistance to chemotherapy in the cases under discussion. Interestingly, EBV-positive breast cancer was correlated with adverse prognostic factors, such as tumor size, axillary lymph node metastasis, vascular invasion, Ki-67 index, tumor stage, loss of estrogen receptor and progesterone receptor expression, and higher PD-1/PD-L1 expression, compared to the EBV-negative group [78]. Also, in this study, survival analysis showed that EBV was associated with poor disease-free survival and overall survival [78].

## 5. Conclusions

In summary, we have described two cases of EBV-associated breast cancer with a lack of response to chemotherapy, suggesting a possible role of EBV in the progression of disease and therapy resistance. This may offer novel diagnostic aspects in the clinical interpretation of breast cancer progression.

## Figures and Tables

**Table 1:** Clinical course of case 1.

	ONCOLOGIC EVENTS	THERAPEUTIC APPROACHES
	56-year-old female patient with the primary diagnosis of a well-differentiated, non-keratinizing, EBV-positive nasopharyngeal carcinoma with cervical lymph node metastases. *Clinical tumor stage:* cT2, cN3b, cM0, G1.	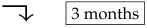 Induction therapy (3 cycles of cis-platin/ docetaxel/5-fluorouracil). Radiochemotherapy with cis-platin. 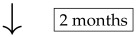 Radiochemotherapy with cis-platin.
5 months a.p.d. *	Near-complete regression of the nasopharyngeal carcinoma and cervical cervical lymph node metastases.	
9 months a.p.d.	Detection of right axillary lymph node metastases and bone metastases by PET-CT.	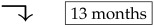 Treatment with 17 cycles of pembrolizumab as immunotherapy, without response.
15 months a.p.d.	Detection of a hypermetabolic spherical mass of the right breast, right axillary lymph node, and bone metastases by CT. *Histopathologic diagnosis on breast core needle biopsy:* triple negative, poorly differentiated invasive breast cancer of no special type (NST, G3; ER-/PR-/HER2-). *Clinical tumor stage: cT3, cN3, M1.*	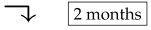 Systemic chemotherapy with paclitaxel/bevacizumab (3 cycles).
16 months a.p.d.	Systemic progressive disease with the formation of diffuse bone metastases and cervical, axillary, infraclavicular, mediastinal, hilar, retroperitoneal, mesenteric, and retrocrural lymph node metastases.	
18 months a.p.d.	Presentation of a mixed response concerning the generalized cancer disease in CT.	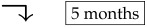 Systemic chemotherapy with 6 cycles of carboplatin/ gemcitabine.
25 months a.p.d.	Mastectomy and axillary lymph node dissection performed as a debulking operation. *Histopathologic tumor stage:* ypT3, ypN2a (9/9), L1, R0. Positive result of the in situ-hybridizsation by EBER-ISH on the resected tumor tissue.	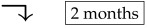 Systemic therapy by 6 cycles of carboplatin/gemcitabine. Experimental vaccination with two peptides of tumor-associated antigens Muc1 and TP53.
Few months.	Death of the patient due to further progressive disease with a lack of response to chemotherapeutic approaches.	

* after primary diagnosis.

**Table 2:** Clinical course of case 2.

	ONCOLOGIC EVENTS	THERAPEUTIC APPROACHES
	49-year-old female patient with the diagnosis of an advanced, lymph node-positive moderately differentiated invasive breast cancer of no special type (NST, G2; immunohistochemically HER2-positive (3+). *Clinical tumor stage:* cT3, cN1, cM0.	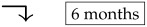 Systemic neoadjuvant therapy with 6 cycles of docetaxel/carboplatin in combination with trastuzumab/ pertuzumab as neoadjuvant systemic therapy.
5 months a.p.d. *	Clinically stable disease. *Pre-operative, post-neoadjuvant clinical tumor stage:* ycT3(sat), ypN1. Tumor resection by quadrantectomy. *Histopathologic tumor stage:* ypT3, ypN1a(3/21), L1, R0. Due to the lack of clinical and pathological response to chemotherapeutic approach, EBV was tested, with serological detection of a highly positive status of latent EBV- infection. The in situ-hybridizsation by EBER-ISH on the resected tumor tissue was also positive.	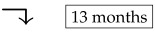 Post-neoadjuvant systemic therapy with 6 cycles of trastuzumab emtansine. Further 8 cycles are planned for further months (ongoing).
12 months a.p.d.	The patient is alive with stable disease.	

* after primary diagnosis.

## Data Availability

Further information, such as laboratory procedures, supporting reported results can be requested to authors.

## Abbreviations

The following abbreviations are used in this manuscript:

BARF1	BamHI-A rightward frame 1
BC	breast cancer
BCL-2	B-cell lymphoma 2 (Bcl-2) family
EBER-1	Epstein-Barr Early Ribonucleoprotein 1
EBNA1	Epstein–Barr nuclear 8 antigen 1
EBV	Epstein–Barr virus
EDTA	Ethylenediaminetetraacetic acid
HER2	human epidermal growth factor receptor 2
IHC	immunohistochemistry
ISH	In situ hybridization (ISH)
HRP	Horesradish-peroxidase
IARC	International Agency for Research on Cancer
LMP1	Late-membrane protein 1
NACT	Neoadjuvant chemotherapy
NST	Invasive breast cancer of no special type
PD	Progressive disease
PKR	Protein kinase R
TNBC	triple-negative breast cancer
